# A Systematic Review and Meta-Analysis of Nature Walk as an Intervention for Anxiety and Depression

**DOI:** 10.3390/jcm11061731

**Published:** 2022-03-21

**Authors:** Simone Grassini

**Affiliations:** 1 Department of Social Studies, University of Stavanger, 4021 Stavanger, Norway; simone.grassini@ntnu.no or simone.grassini@uis.no; 2 Department of Psychology, NTNU–Norwegian University of Science and Technology, 7034 Trondheim, Norway

**Keywords:** nature, walk, depression, anxiety, intervention, review, meta-analysis

## Abstract

Scientific research has widely examined the therapeutic and health benefits of being in contact with natural environments. Nature walk have been proposed as a cost-effective and inclusive method for successfully exploiting nature for the promotion of health and well-being. Depression and anxiety symptoms have been shown to benefit from nature walk. Despite recent empirical findings published in the scientific literature, a summary quantitative work on the effect of nature walk on depression and anxiety does not yet exist. The present systematic review and meta-analysis quantitatively analyze and qualitatively discuss the studies published on the effect of nature walk on depression and anxiety published during the past decade. A database search as well as snowballing methods were used to retrieve eligible articles. The research question and literature search were based on the Preferred Reporting Items for Systematic Reviews and Meta-Analyses (PRISMA) statement. Based on screening and retrieval processes, seven studies met the eligibility criteria and were then included in the quantitative meta-analysis. Risk of bias (RoB) analysis was used to evaluate the quality of the included studies using the Newcastle–Ottawa Scale. After a qualitative evaluation of the studies, data from six experiments were included in the meta-analysis. The meta-analysis show that nature walk effectively improve mental health. The findings were confirmed for the experiments reporting the quantitative data within groups (pre- and post-test) and between groups (experimental vs. control group).

## 1. Introduction

Depression and anxiety are common mental health issues experienced by individuals across the globe [1], and are responsible for high personal and public costs in modern society [2,3]. For that reason, finding cost-effective solutions to improve mental health has become increasingly important [4].

Nature has often been shown to improve psychological well-being [5,6]. The time spent in contact with nature, performing physical activities, has especially been examined to determine the effectiveness of nature-based exercises as a form of therapeutical intervention for the promotion of mental health [7]. The possibility of using low-intensity physical activity in nature—such as walking in natural settings - to reduce symptoms of depression and anxiety has recently attracted the attention of the research community [8]. During the last decade, several scientific experiments empirically examined the effect of nature walk on anxiety and depression [9,10,11,12,13,14]. Low-intensity physical activity has the advantage of being widely accessible, even to disadvantaged portions of the population, such as the elderly [15], and therefore may constitute a highly inclusive type of nature-based therapy for mental health promotion.

The COVID-19 global pandemic has further increased the rate of mental health problems in the general population [16,17], and the potential of the contact with nature to act as a community-level protective factor against mental health issues has been proposed to reduce the negative side-effects of the stress caused by the pandemic [18].

Knowledge on the value of nature walk for depression and anxiety does not only offer a possible cost-effective intervention to boost mental health, but, additionally, it has the possibility to create social and political incentives for the preservation of threatened ecosystems and offers a basis for the economic development of nature-rich areas [19]. The present systematic review and meta-analysis examines the published literature on the effect of nature walk interventions on depression and anxiety. The cost-effectiveness, accessibility, and practicality of nature walk justifies the focus of the present article.

## 2. Tools and Methods

### 2.1. Protocol

**Figure 1:**
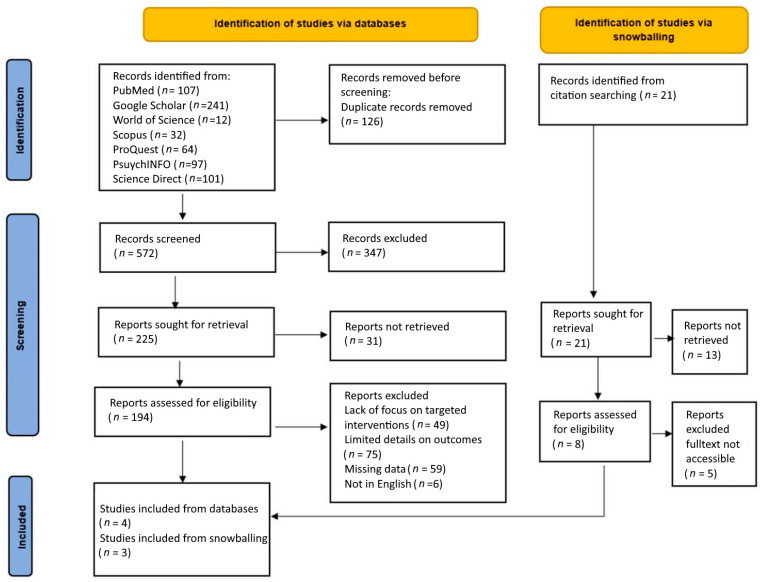
PRISMA diagram.

Recommendations from the Preferred Reporting Items for Systematic Reviews and Meta-Analyses (PRISMA) statement [20] were applied in the present article’s selection and screening process. The author of the present article was responsible for article searching and selection. Keywords and abstracts were screened to determine their suitability for the articles to fulfill the stated research objectives. The initial process involved identifying and excluding the duplicate articles generated across multiple databases. Other subsequent strategies were used to screen, exclude, and include articles to attain the research objectives. A PRISMA flow diagram (Figure 1) is included in the present article to visually describe the various stages of the article selection process.

### 2.2. Search Strategy

Seven online databases were used to search and retrieve the articles that were used in the systematic review and meta-analysis. These databases included were PubMed, Google Scholar, World of Science, Scopus, ProQuest, PsycINFO, and Science Direct. The keywords used in the search were “depression”, “anxiety”, “nature walk”, and “green space”. The study also used reference searching as a snowballing method to find additional articles using as a starting point the recently published systematic review [8].

The search prioritized randomized controlled trials (RCTs), and retrospective and prospective studies. Other studies’ typologies were excluded from the meta-analysis, including qualitative studies, case studies, commentaries, editorial perspectives, systematic reviews, meta-analysis editorial letters, literature reviews, and abstracts.

### 2.3. Eligibility Criteria

The studies considered for inclusion measured the impact of nature walk on depression and anxiety. Studies investigating other forms of walks for which the “nature” component was not predominant, as well as other types of higher intensity physical activities in a natural setting, were excluded. All the studies that were included empirically tested the effectiveness of nature walk to have an impact on the self-reported level of depression and anxiety. Some of the articles included in the review reported data from longitudinal interventions. Others provided a comparative analysis between nature walk and other forms of walks, physical activity, and other types of psychosocial interventions. All the studies that did not report first-hand quantitative data were excluded. Table 1 shows a summary of the inclusion and exclusion criteria used in the present meta-analysis.

### 2.4. Data Extraction

The articles that were duplicated across the databases were excluded. Titles and abstract screening were performed to ensure that the considered articles contributed to answering the objectives of the systematic analysis. The eligibility criteria were applied, and the studies focused on interventions other than nature walk. The articles that did not focus on depression and anxiety, as the outcomes were also excluded. Non-English studies and those that were not RCTs, retrospective studies, or prospective studies were also excluded. The full-text versions of the studies that met the eligibility criteria were explored, and data were extracted for the meta-analysis. A snowball strategy for articles retrieval was used to find additional relevant articles, based on the analysis of relevant citations individuated from the body of scientific literature that fulfilled the inclusion criteria. A table with the characteristics of the included studies is presented (Table 2), with another table providing the empirical findings of the studies (Table 3).

## 3. Results

### 3.1. Study Selection

A total of 654 articles were retrieved from the database search, while 44 were retrieved from the citations of other relevant articles. A total of 572 articles were retained after the removal of the duplicates. From these articles, 225 were retained after title and article screening. The full text for 31 articles was not available.

Additionally, 186 articles were excluded after the full-text screening. Seven articles were ultimately selected for inclusion in the quantitative meta-analysis. The 7 articles included in the present meta-analysis were all published over the last decade, between January 2013 and February 2022. Figure 1 shows the PRISMA diagram illustrating the processes and steps used in screening and exclusion to obtain the final articles used in the analysis. After the data extraction and risk of bias evaluation, one of the seven articles that were ultimately selected was excluded from the meta-analysis [21].

### 3.2. Study Characteristics

All included articles were published between 2013 and 2020. A number of studies [9,10,11,12,13,14] used randomization to assign the participants in the experimental and control groups. The studies included in the analysis were from seven different countries: Netherlands, India, Poland, Korea, Finland, Ireland, and Japan. Other demographic features, such as age and gender, and the type of nature exposure used in these studies are summarized in Table 2.

Seven of the studies examined the effect of nature walk on depression, with three studies [11,13,14] also examining the effect of nature walk on anxiety. One study [12] only examined the effect of nature walk on anxiety. The selected studies implemented different types of questionnaires to assess the anxiety and depression of the participants. The profile of mood status (POMS; [22]) subscales was used by Janeczko et al. [13] and Song et al. [11] to assess both depression and anxiety. The Beck Depression Inventory (BDI; [23]) was used by Korpela et al. [9] and Iwata et al. [21] to assess depression. The Depression Anxiety Stress Scales (DASS; [24]) and the State-Trait Anxiety Inventory (STAI; [25]) were used to measure anxiety.

### 3.3. Outcomes

#### 3.3.1. Pre- and Post- Nature Walk Outcomes

##### Depression

Several studies [9,10,13,14,21] measured the depression indices of the participants before and after the nature walk intervention. Janeczko et al. [13] found that the depression decreased after the nature walk, independently from the type of natural environment experienced in the nature walk.

In the case of Korpela et al. [9], two post-intervention measures at different time-periods resulted in two post-walk outcomes. In the eighth week of the nature walk intervention, the average BDI score for the study participants decreased from before the intervention. In the three-month follow-up period, there was a further decrease. Korpela et al. [9] reported an important reduction in depression during the nature walk intervention, with 46 percent of the participants reporting a clinically significant decrease in depression severity. Gotink et al. [14] also found improvements in the depression of the participants from before to after the nature walk intervention. However, Gotink et al. [14] reported that the depression improvement was moderate overall and was not statistically significant compared to the pre-intervention measure.

##### Anxiety

The study of Janeczko et al. [13] found that anxiety (measured using POMS) decreased after walking in nature, compared to the anxiety reported by the participants before the intervention. Gotink et al. [14] reported moderate and non-significant improvements in the participants’ anxiety across the three periods of measurement. The study of Shin et al. [12] showed anxiety to decrease in different walking conditions; the focus of this analysis was the athletic group walking in forests whose STAI value significantly decreased from 36.3 (SD = 9.3) to 34.6 (SD = 8.1) in the post-walk measures.

#### 3.3.2. Nature Walking and Control Outcomes

##### Depression

The depression subscale of the POMS was used to measure depression in the study by Song et al. [11]. The study showed that there was a significantly lower depression in the nature-walking group (experimental) compared to city-walking group (control). The study of Janeczko et al. [13] showed that all the nature walk conditions improved depression scores (POMS) compared to the control condition (apartment–suburb walks). However, the different types of nature in the three experimental settings (green suburbs, coniferous forests, and deciduous forests) showed similar results and did not differ statistically.

##### Anxiety

Janeczko et al. [13] found that the level of tension was significantly higher in the apartment suburbs than in the other natural conditions. The results further showed the differences across natural conditions, with tension decreasing as one moves from green suburbs to coniferous forests and deciduous forests. The tension–anxiety subscale used by Song et al. [11] showed a significantly lower level of anxiety in the nature-walk group compared to the city-walking one.

### 3.4. Study Quality

The Newcastle–Ottawa Scale [26] was used as a basis to create the RoB to evaluate the quality of the included studies and determine the fit for statistical analysis. The Newcastle–Ottawa Scale was adjusted to fit the types of interventions analyzed in the present a systematic review. The original aspects of the Newcastle–Ottawa Scale referred to as “exposure” and “outcome of interest was not present at the start of the study”, were converted to “intervention” and “measured outcome was assessed before the intervention”, respectively. Representativeness of Exposed Cohort, Selection of Non-Exposed Cohort, Ascertainment of Intervention, Demonstrate Outcome Assessed before Intervention, Comparability of Cohorts on the Basis of Design or Analysis, Assessment of Outcome, Follow-Up Long Enough, Adequacy of Follow-Up, and Missing Data were the areas used to evaluate the studies. The high-risk studies [1,2,3] were excluded from the analysis. All other studies [4,5,6,7,8,9] were included in the statistical analysis. Based on the RoB analysis, one study was excluded for statistical analysis as it was considered as having a high risk of bias [21]. The risk of bias evaluation is reported in Table 4.

### 3.5. Statistical Analysis

**Figure 2:**
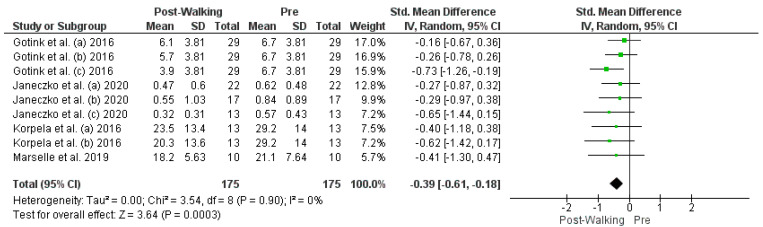
Depression Post- vs. Pre-Nature Walk Forest Plot [9,10,13,14]. Green squares represent standardized mean difference for each study, while black rhombus represents the aggregated average of the standardized mean differences.

**Figure 3:**
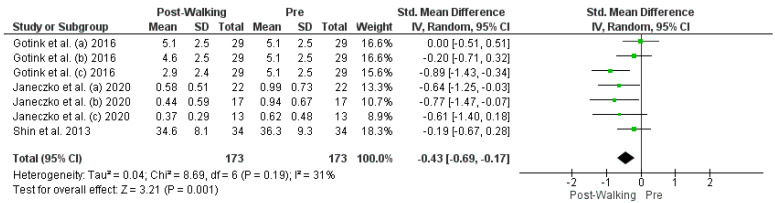
Anxiety Post- vs. Pre-walk Forest Plot [12,13,14]. Green squares represent standardized mean difference for each study, while black rhombus represents the aggregated average of the standardized mean differences.

**Figure 4:**
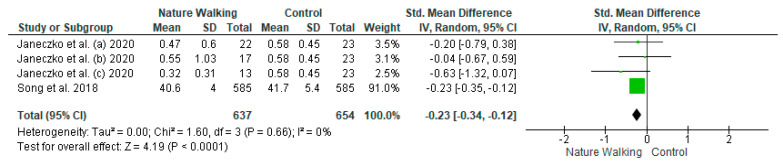
Depression Nature Walk vs. Control Forest Plot [11,13]. Green squares represent standardized mean difference for each study, while black rhombus represents the aggregated average of the standardized mean differences.

**Figure 5:**
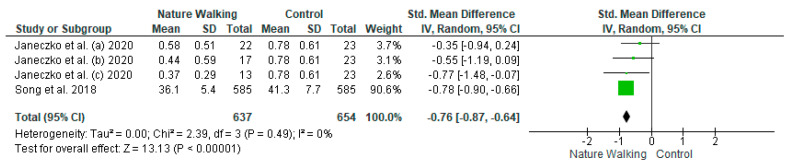
Anxiety Nature Walk vs. Control Forest Plot [11,13]. Green squares represent standardized mean difference for each study, while black rhombus represents the aggregated average of the standardized mean differences.

**Figure 6:**
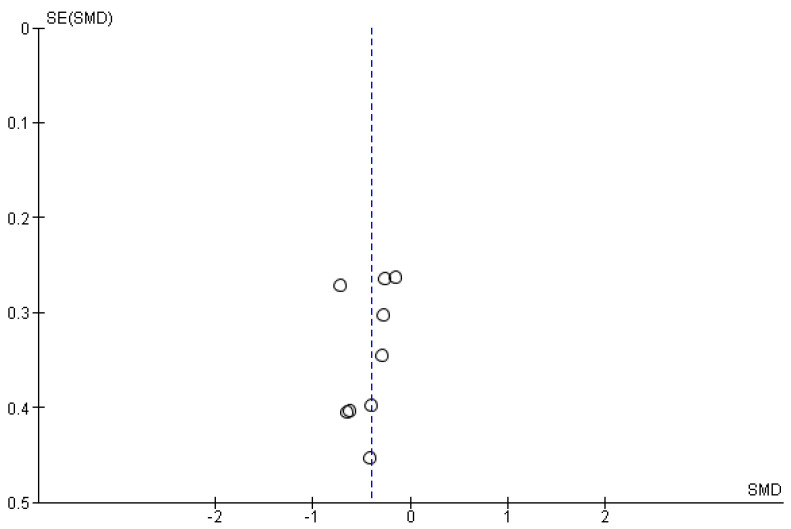
Depression post- vs. pre-nature walk funnel plot.

**Figure 7:**
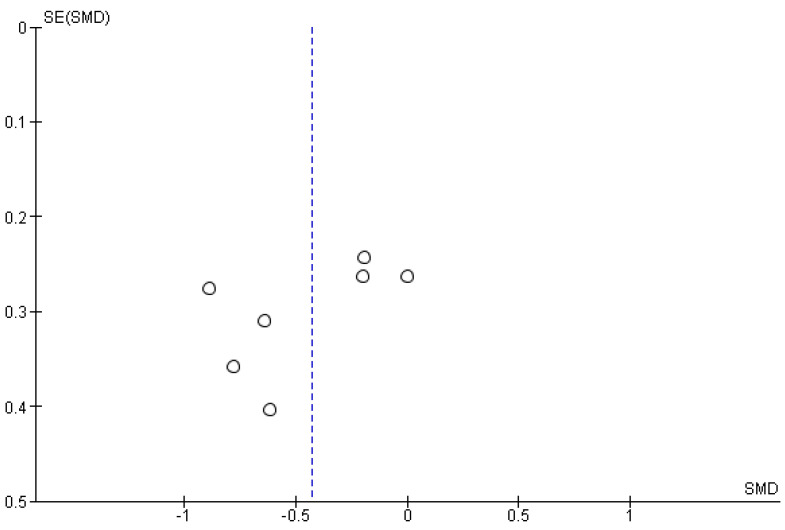
Anxiety post- vs. pre-nature walk funnel plot.

**Figure 8:**
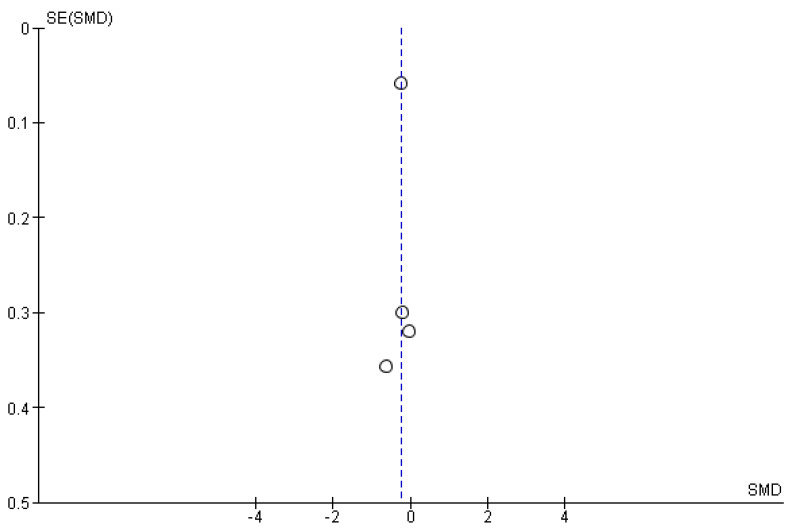
Depression nature walk vs. control funnel plot.

**Figure 9:**
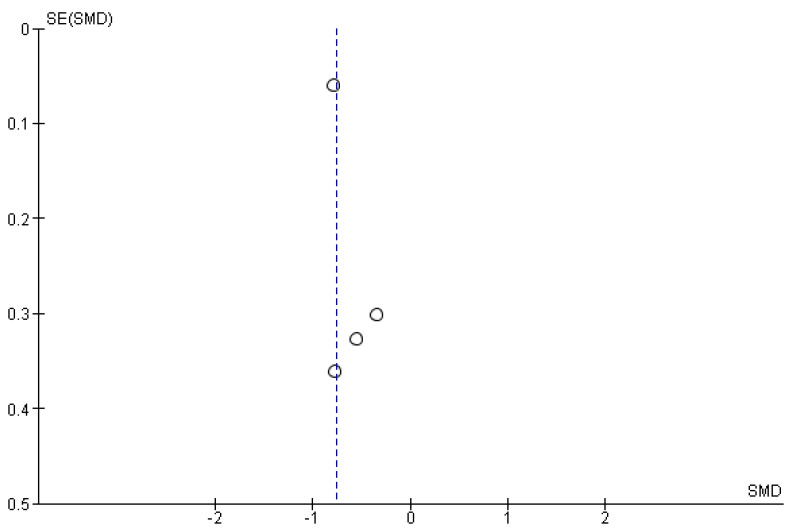
Anxiety nature walk vs. control funnel plot.

The studies deemed fit for analysis after the RoB analysis were pooled together to perform the statistical analysis. When the SD was not provided in the article, it was estimated using the Cochrane Handbook for systematic Reviews of Interventions v. 5.1. In the instances in which the mean was not provided, the median was used. Random effects models were used in the analyses, and forest plots were generated from the standard mean differences of the experimental and control conditions. Two groups of statistical analyses were performed. The first analysis included the means, standard deviations, and totals of the pre- and post-nature walk conditions (Figure 2 and Figure 3). The second analysis was performed to compare the nature-walks condition to the various control conditions reported in the studies (Figure 4 and Figure 5). The *I*^2^ value was computed to determine the heterogeneity. The funnel plots in Figure 6, Figure 7, Figure 8 and Figure 9 is computed to estimate the possible publication biases.

Four forest plots were created from the analyses. The differences in the tools used in measuring depression and anxiety across the studies led to the use of a random-effects approach in the pooling of the studies. The standard mean difference was also used to compare the pre- and post-nature walk outcomes and for the comparison of the nature walk to the control conditions. All the forest plots had a low heterogeneity, with an *I^2^* that was less than 50% (0%, 31%, 0%, and 0%).

All four forest plots showed a significant positive effect of nature walk on improving depression and anxiety symptoms. For the pre-and post-walk forest plots, the depression statistical output of CI = −0.39 [−0.61, −0.18], Z = 3.64 (*p* = 0.0003) and anxiety output of CI = −0.43 [−0.69, −0.17], Z = 3.21 (*p* = 0.001) showed the significant impact of walking on psychological outcomes. The nature walk versus control forest plots had outputs of CI= −0.23 [−0.34, −0.12], Z = 2.18 (*p* < 0.0001) for the depression analysis and CI = −0.23 [−0.87, −0.64], Z = 13.13 (*p* < 0.00001) for the anxiety analysis, thus proving the statistical significance of walking in nature, compared to walking in areas that are not nature-based or have no activity.

## 4. Discussion

According to the results of the present systematic review and meta-analysis, nature walk interventions improve depression and anxiety. The included studies focused on the interventions that provided useful empirical data and solely focused on the impact of nature walk on depression and anxiety. While the two conditions are often reported as co-morbid [27,28], the present meta-analysis separately analyzed the two phenomena. Seven eligible studies analyzing the impact of nature walk on depression and/or anxiety were included in the quantitative meta-analysis. Other than the quantitative presentation of the results of the individual studies, pooling was used to determine the overall effect of all studies.

Nature walks showed to be better than control conditions, such as urban walk, and experiencing of nature without the walking component. Other than Gotink et al. [14], who observed no statistically significant improvements to depression from the pre-walk-to-post-walk conditions, all the other analyzed studies experienced a significant impact from the nature walk. While finding improvements in anxiety levels when comparing anxiety levels before and after the nature-walk intervention, Gotink et al. [14] reported that the changes were moderate and non-significant. The other three included studies that explored the effect of nature walk on anxiety [11,12,13] reported statistically significant improvements to anxiety levels. The heterogeneity in the results across the studies may be attributed to several factors, such as, for example, the use of different measurement tools, and varying the conditions of the nature walk.

Even though depression and anxiety were estimated in the various studies using several different questionnaires, the results for the pooled analysis revealed homogeneity in all the computed forest plots. The pooling of six studies showed that the groups that experienced the experimental nature-walk condition had better psychological outcomes in the post-walk times than the pre-walkg. The study by Iwata et al. [21] lacked the reporting of quantitative information of data distribution, therefore it was excluded from the pooling.

The convenience, availability, and affordability of nature combined with the practical and inclusive nature of walk were identified in the studies as one of the key benefits of the nature walk interventions. The difference between the various types of natural environments has not commonly been investigated with the important exception of the investigation conducted by Janeczko et al. [13]. In a study that included nature walk in green suburbs, coniferous forests, and deciduous forests, the researchers reported that the forest environment had a greater impact on depression and anxiety than the green suburbs. The effect of different types of natural environments on anxiety also varied, with the less dense forests having better outcomes than the dense forests, with such effects being less prominent for depression. These findings suggest that the quality of nature in which nature walk are implemented may be crucial and suggest the importance of considering the quality of natural environments for estimating their possible potential as a clinical intervention tool. The study by Bielinis et al. [29] also showed that the qualitative aspects of natural environments are important for mediating positive health effects.

The experiment conducted by Iwata et al. [21] found that the participants preferred forest walks due to the qualitative features, such as quietness and an almost total absence of people. The break away from a normal routine and the uptake of the freedom and escape provided by the natural settings were also reported to be other attractions towards the intervention. It is possible that these characteristics of natural environments may mediate the positive effects of nature walk when compared to therapeutic-directed walks implemented in urban settings [9,11,13].

With most studies focusing on group walks and none comparing group and individual walks, it may be important for future investigations to examine the impact of social interactions during the nature walk with a focus on health and well-being promotion.

Several limitations can be individuated in the present study. The different tools used to measure depression and anxiety might lead to biased outcomes in the statistical analysis, despite the overall low level of heterogeneity presented in the quantitative data analysis. Future studies should consider pooling studies that use a specific tool to measure psychological outcomes. The methodologies of the included studies are another concern of the quality of the statistical analysis. The lack of follow-ups in most of the studies implied that long-term effects could not be adequately assessed. The lack of randomization and participants/experimenters blinding in the studies might have also contributed to biased results. Future studies investigating nature-based interventions should use experimental designs featuring randomized controlled trials. The lack of consistency in the inclusion criteria of the studies, with some having clinically diagnosed patients and others having severe or moderate anxiety or depression participants, makes it difficult to translate the findings for the general population. Consistencies in the seasons and nature walk interventions of the pooled studies are also crucial in improving the quality of future polling analysis. Contrarily to a recently published article on the same topic [8], the studies included in the present work and the subsequent quantitative analysis show that the scientific literature presents convincing (despite limited in number) evidence for the benefit of nature walk on anxiety and depression. The difference between the present study and the previous published review [8] may be attributable to a difference in the studies included, and from the fact that Kotera et al. [8] admittedly, included studies with broad aims and with high risk of bias (as reported in their RoB). The present review and meta-analysis attempted to evaluate only studies with a more focused aim on health effects of nature walk and exclude studies that were judged as high risk for bias.

Meta-analyses have the potential to discover possible publication bias in the scientific literature and therefore contribute to the ongoing debate on the reproducibility crisis of research in psychology [30]. From the funnel plots that were presented, the data extracted from the studies included in the present meta-analysis did not show a high risk of publication bias (however, see some criticisms on such method for identifying publication bias, as discussed in Zwetsloot et al. [31]).

## 5. Conclusions

The studies included in this analysis assessed the impact of nature walk on anxiety and depression. The systematic review and meta-analysis show that nature walk effectively improve mental health, positively impacting depression and anxiety. The within group and between group results argue in favor of the effectiveness of nature walk. Despite the absence of adequate studies performing follow-ups to help determine the long-term effects, a positive effect of nature walk was reported for up to three months. The current findings are critical in demonstrating the empirical value of nature-based walk interventions for improving mental health.

## Figures and Tables

**Table 1:** Inclusion and exclusion criteria.

Description	Inclusion Criteria	Exclusion Criteria
Intervention	Nature walk	Other forms of walks, physical activities, and interventions
Outcomes	Depression and anxiety levels and symptoms	Other psychological and physical outcomes, excluding either depression or anxiety
Patients	Any population	-
Period	Publication date is during or after 2011	Publication date is before 2011
Language and Format	Full-length English articles	Not full-length or non-English articles
Design of Studies	RCTs, retrospective studies, prospective studies	Qualitative studies, single case studies, commentaries, editorial perspectives, systematic reviews and meta-analysis editorial letters, literature reviews, and abstracts

**Table 2:** Study characteristics.

Author	Year	Country	Sample			Intervention	Measures	Findings
			Description	Mean Age (Years)	Gender			
Janeczko et al. [13]	2020	Poland	A total of 75 university students walking in deciduous forests, coniferous forests, green suburbs, and apartment suburbs			30 min 2 km walk	Profile of Mood States (POMS)	Differences between the groups showed the reduction of depression following the intervention
Song et al. [11]	2018	Japan	A total of 585 male students walking in urban areas or forests	21.7 (1.6)	585 M, 0 F	15 min walk	POMS and State-Trait Anxiety Inventory (STAI)	Nature walks significantly reduced depression, anxiety, and trait anxiety compared to urban walks
Korpela et al. [9]	2016	Finland	A total of 13 clinical depression patients were randomly assigned to urban and nature walk conditions	48 (median 52) (29–59)	4 M, 9 F	2 h/week walks for 8 weeks	Beck Depression Inventory (BDI)	Depression reduced from pre-walk to post-walk period and in the 3-month follow up
Marselle et al. [10]	2019	England	A total of 1516 participants	88% age ≥ 55	34%M:66%F	A 13-month nature walk intervention. Participants in group walking and non-walking conditions	A 10-item major depressive inventory	A greater benefit on depression observed for the walking group compared to the non-walking group
Iwata et al. [21]	2016	Ireland	A total of 15 clinical patients	47 (32–72)	3 M:12 F	2 h/week walks for 13 weeks. A total of 10 min warm-up, 1–1.5 h forest walk, 30 min refreshments in the forest	Hamilton Depression Rating Scale (HDRS) and BDI	The levels of depression significantly lowered in the HDRS (11.84–5.98) and BDI (36.8%) after exercise
Gotink et al. [14]	2016	Netherlands	A total of 29 participants	54.3 (9.0)	31%M:69%F	A 1-day walking retreat in a group (accompanied by a mindfulness teacher), 3-day walking retreat in a group (accompanied by 2 mindfulness teachers), 6 days + solitary walking retreat	The Dutch version of the Depression Anxiety Stress (DASS-21)	Improvements to depression levels, however not statistically significant
Shin et al. [12]	2013	Korea	A total fo 139 participants	18–25	0 M, 139 F	Athletic walking in the gymnasium (AG) group, athletic walking in the forest (AF) group, meditative walking in the gymnasium (MG) group, and meditative walking in the forest (MF) group	State-Trait Anxiety Inventory-X	Meditative walking had a more significant effect on depression than athletic walking

**Table 3:** Study characteristics (empirical findings of the studies).

Author	Description	Psychological Outcome	Psychological Outcome	Walking			Control			Pre walking		
				Mean	SD	Total	Mean	SD	Total	Mean	SD	Total
Janeczko et al. [13]	Green suburbs	Depression	POMS	0.47	0.6	22	0.58	0.45	23	0.62	0.48	22
	Coniferous forest	Depression	POMS	0.55	1.03	17				0.84	0.89	17
	Deciduous forest	Depression	POMS	0.32	0.31	13				0.57	0.43	13
	Green suburbs	Anxiety	POMS	0.58	0.51	22	0.78	0.61	23	0.99	0.73	22
	Coniferous forest	Anxiety	POMS	0.44	0.59	17				0.94	0.67	17
	Deciduous forest	Anxiety	POMS	0.37	0.29	13				0.62	0.48	13
Song et al. [11]	Experimental and control groups	Depression	POMS	40.6	4	585	41.7	5.4	585	-	-	-
	Experimental and control groups	Anxiety	POMS	36.1	5.4	585	41.3	7.7	585	-	-	-
Korpela et al. [9]	After 8 weeks,	Depression	BDI	23.5	13.4	13	-	-	-	29.2	14	13
	3-month follow-up	Depression	BDI	20.3	13.6	13	-	-	-			
Marselle et al. [10]	Group walk in nature	Depression	A 10-item major depressive inventory	−0.08	0.02	1506	-	-	-	0.48	0.02	1506
Iwata et al. [21]	Pre-walk and post-walk	Depression	BDI	14.93	-	15	-	-	-	22.86	-	15
Gotink et al. [14]	Pre-walk and postwalk (time 1)	Depression	DASS-21	6.1	3.81	29	-	-	-	6.7	3.81	29
	Pre-walk and post-walk (time 2)	Depression	DASS-21	5.7	3.81	29	-	-	-			
	Pre-walk and post-walk (time 3)	Depression	DASS-21	3.9	3.81	29	-	-	-			
	Pre-walk and post-walk (time 1)	Anxiety	DASS-21	5.1	2.5	29	-	-	-	5.1	2.50	29
	Pre-walk and post-walk (time 2)	Anxiety	DASS-21	4.6	2.5	29	-	-	-			
	Pre-walk and post-walk (time 3)	Anxiety	DASS-21	2.9	2.4	29	-	-	-			
Shin et al. [12]	Pre-walk and post-walk	Anxiety	STAI	34.6	8.1	34	-	-	-	36.3	9.3	34

**Table 4:** Risk of Bias (RoB). The * symbol mean that the study is fulfilling the criteria.

	Representativeness of Exposed Cohort	Selection of Non-Exposed Cohort	Ascertainment of Intervention	Demonstrate Outcome Assessed before Intervention	Comparability of Cohorts on the Basis of Design or Analysis	Assessment of Outcome	Follow-Up Long Enough	Adequacy of Follow-Up	Data available (No Missing Data)	Total
Janeczko et al. [13]	*	*	*	*		*			*	6
Song et al. [11]		*	*		*	*			*	5
Korpela et al. [9]			*	*		*	*	*	*	6
Marselle et al. [10]	*	*	*		*	*			*	6
Iwata et al. [21]			*	*		*				3
Gotink et al. [14]	*	*	*		*	*			*	6
Shin et al. [12]	*		*		*	*			*	5
